# A Bi‐Gradient Dielectric Polymer/High‐*Κ* Nanoparticle/Molecular Semiconductor Ternary Composite for High‐Temperature Capacitive Energy Storage

**DOI:** 10.1002/advs.202302949

**Published:** 2023-07-14

**Authors:** Manxi Li, Yujie Zhu, Rui Wang, Jing Fu, Zhaoyu Ran, Mingcong Yang, Junluo Li, Jun Hu, Jinliang He, Qi Li

**Affiliations:** ^1^ State Key Laboratory of Power Systems Department of Electrical Engineering Tsinghua University Beijing 100084 China

**Keywords:** energy density, high‐temperature capacitors, molecular semiconductors, phase‐field simulation, polymer dielectric

## Abstract

Attaining compact energy storage under extreme temperature conditions is of paramount importance in the development of advanced dielectric materials. The polymer composite approach has proved effective towards this goal, and addressing the correlation between filler distribution and electrical properties is foremost in designing composite dielectrics, especially in multifiller systems. Here, the design of a bi‐gradient polymer composite dielectric using an integrated framework based on the phase field model is reported. This framework can predict the charge‐inhibiting behavior of composite dielectrics, which is a key factor impacting the high‐temperature capacitive performance but unfortunately is ignored in conventional phase field models. It is found that due to the traps provided by the functional organic fillers, more carriers are trapped near the electrodes and weaken the electric field, thus significantly suppressing the breakdown initialization process. An interpenetrating gradient structure is designed rationally and synthesized experimentally, which exhibits concurrent high energy density (5.51 J cm^−3^) and high charge‐discharge efficiency (90%) up to 200 °C. This work provides a strategy to predict the high‐temperature energy storage performance of polymer composites containing charge‐inhibiting components and helps broaden the scope of data‐driven materials design based on phase‐field modeling.

## Graphical abstract

A strategy to predict the high‐temperature energy storage performance of polymer composites containing charge‐inhibiting components is provided. An interpenetrating gradient structure of dielectric polymer/high‐dielectric nanoparticle/molecular semiconductor is designed rationally and synthesized experimentally, which exhibits concurrent high energy density of 5.51 J cm^−3^ and high charge–discharge efficiency of 90% up to 200 °C.

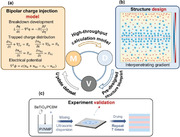

## 1 Introduction

Polymer‐based dielectric film capacitors have received increasing attention on account of their high voltage endurance, fast charge‐discharge rate, low power loss, and graceful failure, giving rise to superior energy storage property and great reliability.^[^ ^1^, ^2^, ^3^, ^4^ ^]^ The lightweight, low cost, and facile processability of the dielectric polymers allow large‐scale industrial applications.^[^ ^5^, ^6^, ^7^, ^8^, ^9^ ^]^ Recently, with rapidly‐increasing demand for compact dielectric energy storage in hybrid electrical vehicles, aerospace power electronics, wind generators, and downhole gas explorations, it has become vitally important for dielectric polymers to possess high discharged energy density (*U*_e_) under elevated temperature conditions.^[^ ^10^, ^11^ ^]^ This yet remains extremely challenging not only because of the dilemma of inversely correlated dielectric constant (*κ*) and breakdown strength (*E*_b_), but also due to the fact that a considerable amount of stored electrical energy (*U*_s_) would dissipate as Joule heat at high temperature, leading to thermal runaway and catastrophic breakdown of the device.^[^ ^11^, ^12^, ^13^ ^]^ Generally, *U*_e_ of the dielectric material can be calculated with the applied electric field (*E*) and the induced electrical displacement (*D*) (see Supporting Information Figure S1, Supporting Information for detail). Particularly, for a linear dielectric, the stored energy density can be expressed as *U*_s_ = 1/2*ε*_0_ *κE*_b_^2^, where *ε*_0_ is the vacuum permittivity, and the maximum energy density is achieved at the breakdown strength *E*_b_ of the dielectric material.^[^ ^14^, ^15^, ^16^, ^17^ ^]^ Apparently, achieving high energy density calls for concomitant enhancement of *κ* and *E*_b_.^[^ ^18^ ^]^ In addition, *U*_e_ also critically depends on the charge‐discharge efficiency *η*, represented by (*U*_e_/*U*_s_) × 100% (Figure S1, Supporting Information). Under high‐temperature conditions, the efficiency is usually significantly lower than that at room temperature, owing to the various temperature‐ and field‐dependent conduction mechanisms that generate energy loss in the form of Joule heat.

To attain compact dielectric energy storage under extreme temperature conditions, we propose to concurrently introduce high‐*κ* ceramic nanoparticles^[^ ^19^, ^20^, ^21^, ^22^, ^23^ ^]^ and molecular semiconductors^[^ ^24^, ^25^ ^]^ into the polymer matrix to increase the dielectric constant and impede the charge transport (conduction loss), respectively. In such a complex dielectric polymer/high‐*κ* nanoparticle/molecular semiconductor ternary composite, the correlation between the filler distribution and electrical properties is a critical issue. In previous research, a comprehensive electric‐thermal‐mechanical phase‐field model has been developed to simulate the charge response and dielectric breakdown evolution in polymer/high‐*κ* inorganic nanoparticle composites, and composite structures with high energy storage density have been designed via the machine learning method.^[^ ^26^, ^27^, ^28^ ^]^ Nevertheless, this model does not apply to the composites doped with organic fillers for charge regulation, which is a key factor impacting the high‐temperature capacitive performance.

In this paper, we put forward an integrated framework based on the phase field method for the design of dielectric polymer composite containing complex organic/inorganic fillers with excellent high‐temperature energy storage performance. Space charges are introduced in the simulation of breakdown evolution to describe trap effects brought by the organic molecular semiconductor. The coupling simulation of charge behavior and breakdown evolution is performed, where various charge events are taken into account, including the injection, trapping, de‐trapping, and combination of the free electrons, the free holes, the trapped electrons, and the trapped holes.^[^ ^29^ ^]^ Based on the comprehensive framework, a bi‐gradient organic/inorganic composite structure is proposed, and high‐throughput computational calculations are performed to systematically explore the structure‐property correlation in the PI‐based composite. Guided by the calculation results, the structure is further optimized.^[^ ^30^ ^]^ We prepare a seven‐layer PI/BT/PCBM (polyimide/barium titanate/[6,6]‐Phenyl C61 butyric acid methyl ester) composite film with interpenetrating gradient structure and obtain a giant discharged energy density above 90% efficiency of 5.51 J cm^−3^ at 200 °C, which not only far outperforms that of the pure PI (2.8 J cm^−3^), but also is higher than many of the current dielectric polymer composites under the same temperature condition.^[^ ^9^ ^]^ The framework built in this work offers theoretical guidance on the rational design of a wide range of polymer composite dielectrics containing complex organic or inorganic fillers. It provides a solution about how traps affect breakdown strength in phase‐field modeling, and the generality of this approach is verified.

## 2 Results and Discussion

Recently, introducing functional organic fillers with high electron affinity into polymers has become a popular method, which is beneficial for creating deeper trap, restricting carrier transport, and reducing conduction loss.^[^ ^24^, ^25^, ^31^, ^32^, ^33^ ^]^ But very few studies have been reported on the relationship between the distribution of organic fillers and breakdown processing. In previous models describing the breakdown evolution, the driving forces of the order parameter evolution mainly originate from the electric term caused by the different *κ* between inorganic fillers and matrix,^[^ ^26^, ^34^ ^]^ and the Joule heat caused by electrical conduction at high temperature.^[^ ^27^, ^28^, ^35^ ^]^ Taking the molecular semiconductor as an example, the previous model would consider it as an accelerating factor for the breakdown phase growth because of its higher electrical conductivity and consequently the higher thermal energy in comparison to the polymer matrix (see Supporting Information for detail), rather than improving spatial charge distribution. Although the dynamic evolution of charge transport has been realized,^[^ ^36^ ^]^ the coupling of charge transport and breakdown evolution has not been incorporated into a model.

**Figure 1:**
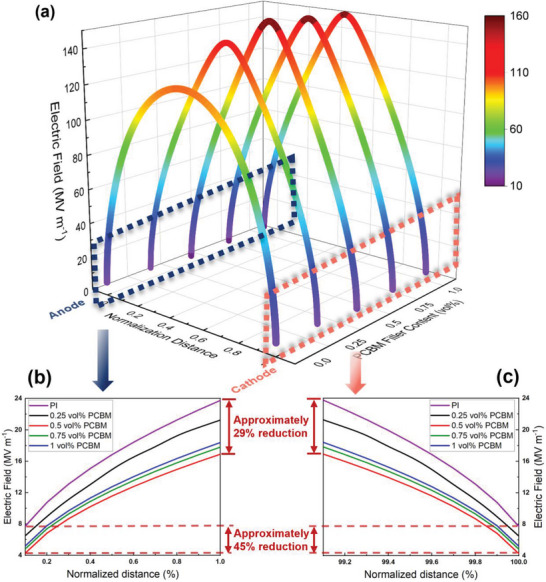
Electric field distribution of a) the overall spatial, magnified part near b) the anode and c) the cathode in homogeneous PI‐based composites loaded with different contents of PCBM (from 0 vol% to 1 vol%) obtained by simulation of bipolar charge injection model. The applied electric field is 100 MV m^−1^.

In view of these problems, the qualitative correlation between traps and breakdown strength is a key step towards building quantitative models for breakdown strength of organic composite dielectric. Here, the key charge transfer kinetic parameters are first deduced based on a literature method ^[^ ^37^ ^]^ and further used in the continuum phase‐field model to capture the space charge and predict the breakdown strength. We take the PI/PCBM composite as an example to expound the modified model. Given the small size (radius ≈ 10 Å) and the uncertain topological structure of doped semiconductor molecules inside the matrix, it is unreasonable to model the organic fillers as fixed particles with specific morphology in the phase‐field modeling on the nanoscale simulation step size.^[^ ^38^, ^39^, ^40^ ^]^ The polymer/molecular semiconductor composite is viewed as a homogeneous domain and the traps are considered to be uniformly distributed in the composite. The depth and density of deep traps in the composite are calculated from the thermally stimulated discharge current test results using the half‐peak method after peak fitting,^[^ ^41^ ^]^ as displayed in Figure S3 and Table S1, Supporting Information. The simulated electric field distribution of the composites with different PCBM concentrations is shown in **Figure** **1**a. Taking into account the hindering effect of deep traps on carriers, the electric field changes from uniform distribution to the “arch bridge” distribution, where it is very small around the electrodes and gets larger in the center. This is attributed to the fact that substantial carriers are injected into the dielectric after overcoming the Schottky energy barrier, and are captured by traps, thus generating carrier shields and reducing the interfacial electric field near the electrode/dielectric interface. The subsequent injection of charges will be suppressed further, and the evolution of breakdown phase is impeded. Due to the unbalance between the speed of charge injection on the boundary and charge propagation in the domain, huge amount of the carriers will be stuffed up around the electrodes. The trapped carriers then weaken the boundary injection electrical field until the process of injection and propagation reaches a dynamic balance according to Poisson's equation. Benefitting from the deep traps brought by molecular semiconductors, the electric field of PI/0.5 vol % PCBM composite is 45% smaller than that of pure PI near the electrodes (Figure 1b,c). According to Table S2, Supporting Information, when the concentration of doping molecules increases, the depth of deep traps basically remains fixed, and the trap density increases first and then decreases. The introduction of high‐electron‐affinity molecular semiconductors brings deep traps at low filler content. At higher filler content, however, the fillers agglomerate and the specific surface area decreases, hence decreasing the deep trap density. Thus, we can map the density of deep traps to the concentration of organic fillers, which is highly related to the space charge and electric field distribution, and will critically affect the evolution of the phase value.

**Figure 2:**
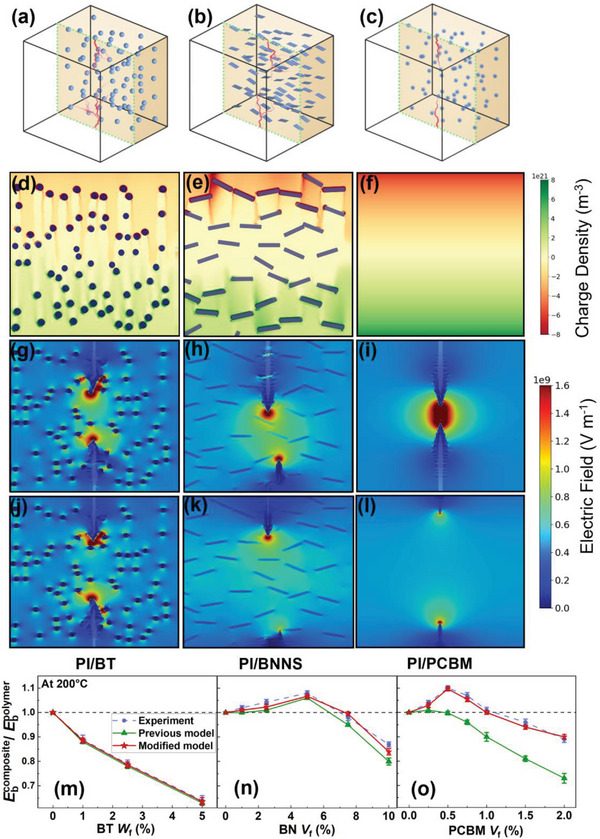
The charge injection effect on the breakdown process of polymer composites. 3D microstructural diagrams and breakdown phase evolutions of PI‐based composites by phase field simulation with a) BTs, b) parallel BNNSs and c) PCBM at optimal volume fraction of 1 wt.%, 5 vol% and 0.5 vol%, respectively. d–f) The distributions of trapped charge density. Corresponding 2D‐sliced breakdown paths along the cross‐section g–i) without and j–l) with charge injection at 500 MV m−1 and 200 °C by phase field simulation. m‐o) Comparison of the experimental values, primary and modified phase field simulation results of the breakdown strength of three dielectric composites.

In order to verify the generality of the model, we select three representative fillers: BT nanoparticles with high dielectric constant, BNNSs (boron nitride nanosheets) with high aspect ratio, and molecular semiconductors (PCBM) with high electron affinity. **Figure** **2**a–c shows the PI‐based composites containing these three kinds of fillers, respectively. Figure 2d–f demonstrates the pure charge density (*n*_pure_ = *n*_free_hole_ + *n*_trapped_hole_ + *n*_free_electron_ + *n*_trapped_electron_) for the three composites at the optimal compositions. Owing to the large mismatch of dielectric constant between BT nanoparticles and the matrix, aggregation of charges occurs at interfacial regions. For PI/BNNS, carriers are obstructed by the high aspect ratio of BNNSs and aggregate at the edge of the fillers. It is observed that more carriers are trapped around the electrodes in the deep traps brought by molecular semiconductors in PI/PCBM composite compared with PI/BT and PI/BNNS. The breakdown processes for the three composites under applied electric field without and with charge injection at 500 MV m^−1^ and 200 °C are respectively displayed in Figure 2g‐i,j‐l. In comparison to the conventional method, the breakdown processes of PI/BT and PI/BNNS are slightly slowed down to varying degrees, depending on the deep traps they introduce (Figure 2j,k). As for PI/PCBM, the development of phase variables in scenarios with traps is suppressed at the initial stage, and accelerates when it progresses to the middle area with or without the charge injection. The phase variable in Figure 2l is only one‐third of that in Figure 2i under the same time unit. In the modified model, abundant traps brought by the organic fillers increase the breakdown field strength at a low filling amount. Laboratory experiments are carried out and the comparison between the two models and experimental results is displayed in Figure 2m‐o. The measured breakdown strengths of the three composites exhibit different trends depending on the doping contents, but all of them are in approximate agreement with the simulation results of the modified model. The comparison shows that the modified model not only predicts the breakdown strength of polymer/inorganic filler composite accurately, but also it is applicable to the prediction of polymer/organic molecule composite.

**Figure 3:**
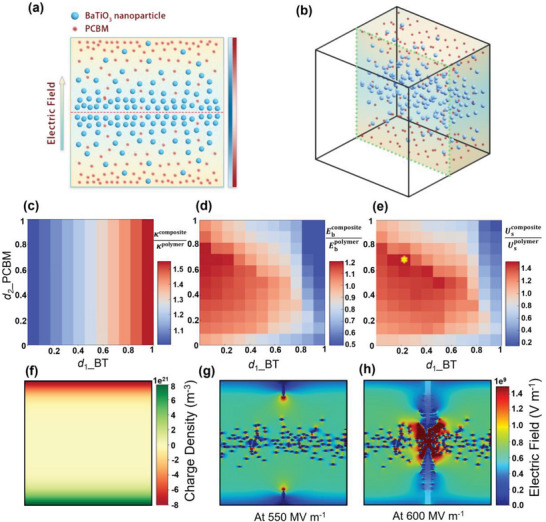
A designed composite based on modified phase‐field model and high throughput computation. a) 2D and b) 3D schematic microstructure of the composite. The phase diagrams of c) the effective dielectric constant, d) the breakdown strength, and e) the energy density compared to a pure polymer by the high throughput calculation as *d*_1_ and *d*_2_ vary. f) The trapped charge distribution and the breakdown paths of the optimal microstructure with *d*_1_ = 0.2 and *d*_2_ = 0.7 at 200 °C with applied electric field of g) 550 MV m^−1^ and h) 600MV m^−1^.

High energy density can be achieved through synergistic enhancement of *κ* and *E*_b_. For example, sandwich structures are designed, where the upper and the lower layers are filled with high‐*E*_b_ or high‐insulation fillers, and the middle layer is filled with high‐*κ* fillers.^[^ ^43^, ^44^, ^45^ ^]^ Here, a design strategy with interpenetrating gradient structure is proposed. PCBM fillers are incorporated in PI with gradient‐structure, where the content decreases linearly from the surface (0.75 vol%) to a certain position of the films. And BTs are placed with reverse gradient‐structure, where the content decreases linearly from the center (5 wt.%) to a certain position of the films. Therefore, an interpenetrating gradient‐structure of PI/PCBM/BT composites is formed, as illustrated in **Figure** **3**a,b. According to the simulation results of the modified phase‐field modeling, placing PCBM only near the electrodes can effectively adjust the trap distribution and avoid the increase in electrical conductivity caused by rising content. On the other hand, placing BTs only in the central region can increase the *κ* of the whole system and avoid accelerating the initial development of breakdown phase at the electrodes. The study is focused on half the plane with symmetric distribution of fillers by controlling the length ratios of BT *d*_1_ and PCBM *d*_2_, where *d*_1_ and *d*_2_ are the opposite normalized distance along the out‐of‐plane direction with opposite gradient, representing the distribution range of the two fillers (Figure S4, Supporting Information). The BT's mass fraction *f*_BT_ at a certain place *d*_BT_ (the distance from the center, where 0 ≤ *d*_BT_ ≤ *d*_1_) along the out‐of‐plane direction follows a linear gradient function, expressed as *f*_BT_ = 5 – 5*d*_BT_ (wt.%). In proportion, a reverse gradient structure for the PCBM volume fraction follows *f*_PCBM_ = 0.75 – 0.75*b* (vol%), where *f*_PCBM_ represents the PCBM's mass fraction at a certain place *d*_PCBM_ (the distance from the surface, where 0 ≤ *d*_PCBM_ ≤ *d*_2_) along the out‐of‐plane direction.

High throughput calculations are performed to systematically generate a structure‐property database and investigate the optimal microstructure for high energy density comprehensively. To address the influence of intrinsic properties of fillers on *E*_b_ and *κ* of various microstructures, we set up a database (11 × 11) of the composites by implementing the modified phase‐field modeling, as shown in Figure 3c–e. Simulations of the 121 structures are operated by assigning different *d*_1_ and *d*_2_. The trap depth and density are obtained by interpolation method and *κ* is calculated from the effective medium theory. As the mapping of *κ* ^composite^/*κ* ^polymer^ displayed in Figure 3c, *κ* ^composite^ increases rapidly with the increase of *d*_1_, while *d*_2_ shows little effect on *κ* ^composite^. For *E*_b_^composite^ in Figure 3d, however, the variation pattern is quite different: higher *d*_1_ leads to a decrease in *E*_b_^composite^ when *d*_2_ remains constant. At the same time, when *d*_1_ is constant, *E*_b_^composite^ first increases and then decreases with the increase of *d*_2_. The increase is explained by the introduction of deep traps and improved electric field distribution. The decrease occurs because of the growing electrical conductivity and aggregation caused by high concentration, especially when the heating area (PCBM) crosses the electric field distortion area (BT), i.e., *d*_1_ + *d*_2_ > 1, *E*_b_^composite^ drops sharply. The maximum value of *E*_b_^composite^ reaches 714 MV m^−1^ where *d*_1_ = 0 and *d*_2_ = 0.75. With the values of *E*_b_ and *κ*, the energy density is calculated by the approximation *U*_s_ = 1/2*ε*_0_ *κE*_b_^2^. As shown in Figure 3e, with the increase of *d*_1_ and *d*_2_, *U*_s_ goes down from the left upper to the right bottom of the mapping and reaches its maximum value when *d*_1_ = 0.2 and *d*_2_ = 0.7, which is about 1.58 times that of the polymer matrix. The charge distribution and breakdown path corresponding to the optimal structure are shown in Figure 3f–h.

**Figure 4:**
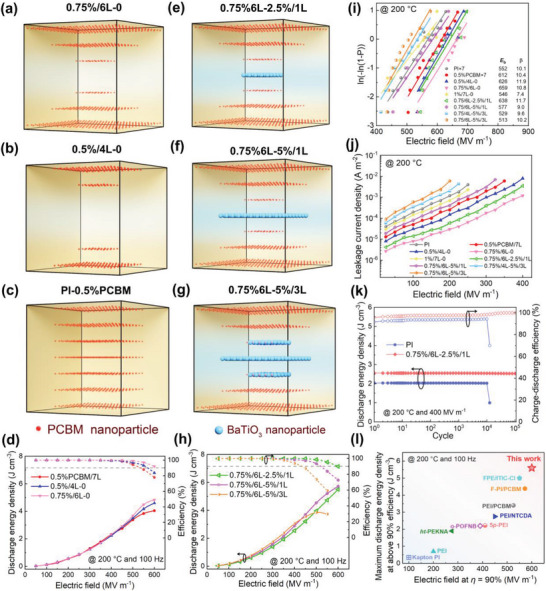
a–h) Structure diagrams and corresponding energy storage characteristics at 200 °C and 100 Hz of PI/PCBM/BT composites with different distributions: 0.75%6L‐0, 0.5%/4L‐0, 0.5%PCBM, 0.75%/6L‐2.5%/1L, 0.75%/6L‐5%/1L, 0.75%/6L‐5%/3L. i) Two‐parameter Weibull distribution analysis of the breakdown strength at 200 °C. j) Electric field‐dependent leakage current density at 200 °C. k) Cyclic stability of PI and the 0.75%/6L‐2.5%/1L composite at 200 °C and 400 MV m^−1^. l) Comparison of the maximum discharged energy density at *η* > 90% of 0.75%/6L‐2.5%/1L and current polymer dielectrics at 200 °C and 100 Hz.

Under the guidance of the calculation results, we construct an interpenetrating gradient structure of PI/PCBM/BT ternary dielectric composite, and the multilayer films are prepared through a layer‐by‐layer solution method by tuning the volume fraction of BT and PCBM particles in each layer. More details about the experimental process and characterization are displayed in Supporting Information. The multilayer film is named *x* %/*a* L‐*y* %/*b* L, where *x* and *y* are the concentration of PCBM at electrodes and BT at center, *a* and *b* represent the number of layers of PCBM and BT. With the central layer as the axis of symmetry, the concentration intervals of PCBM and BT between adjacent layers are fixed as 0.25 vol% and 2.5 wt% respectively. For example, a 7‐layer composite with the contents of PCBM and BT 0.75 vol%/0, 0.5 vol%/0, 0.25 vol%/5 wt.%, 0/2.5 wt.%, 0.25 vol%/5 wt.%, 0.5 vol%/0 and 0.75 vol%/0, respectively from the first layer to the seventh layer is termed as 0.75%/6L‐5%/3L for short. According to the calculation results in **Figure** **4**e, the structure of 0.75%/6L‐2.5%/1L has the highest energy density under the set experimental gradient.

To verify the enhancement effect on breakdown strength of the gradient distribution, 1%/7L‐0 (Figure S10, Supporting Information), 0.75%/6L‐0 (Figure 4a), 0.5%/4L‐0 (Figure 4b), and the even‐distributed composite (Figure 4c) are prepared for comparison. The electrical displacement‐electric field (*D*‐*E*) loops of the composites are measured and given in Figure 4d and Figure S10, Supporting Information. 0.75%/6L‐0 demonstrates the best energy storage performance with the discharge energy density of 4.83J cm^−3^ and efficiency of 92% at 600 MV m^−1^ and 200 °C. And the *E*_b_ increases from 552 MV m^−1^ for PI to 659 MV m^−1^ for 0.75%/6L‐0 (Figure 4i). Then BTs are added on the basis of 0.75%/6L‐0, as shown in Figure 4e–h and Figure S10, Supporting Information. The resulting 0.75%/6L‐2.5%/1L obtains the highest discharged energy density of 5.51 J cm^−3^ with the efficiency over 90%. It manifests 90% enhancement in comparison to the 2.8 J cm^−3^ of PI, and 49% enhancement in comparison to the 3.7 J cm^−3^ of the composite with 0.5 vol% PCBM randomly distributed. And the *E*_b_ only slightly reduces from 659 MV m^−1^ to 638 MV m^−1^. This indicates that the designed bi‐gradient distribution increases the dielectric constant of the composite effectively without significantly sacrificing the breakdown strength, achieving the trade‐off between *E*_b_ and *κ*. It is noteworthy that although sample 0.75%/6L‐2.5%/3L reaches higher discharge energy density over all others due to higher *κ*, its *η* is slightly decreased because of the conduction loss brought by BT. The leakage current in Figure 4j shows that the gradient distribution of molecular semiconductors is more effective in immobilizing carriers and suppressing the leakage current. Moreover, the designed structure exhibits marginal impacts on the dielectric constant and dissipation factor of the polymer composites (Figure S11, Supporting Information). The composite also outperforms the pristine PI in cyclic charge‐discharge tests, indicating better long‐term stability and reliability at high temperature and high electric field (Figure 4k and Figure S12, Supporting Information). Notably, it is clearly seen that the bi‐gradient composite outperforms the previous dielectric composites at 100 Hz (Figure 4l).^[^ ^26^, ^31^, ^33^, ^47^, ^48^, ^49^ ^]^ The designed gradient distribution structure demonstrates remarkable superiority compared to other composites with random distribution, and the modified phase‐field model is also verified.

## 3 Conclusion

In this work, an integrated framework with charge behavior has been proposed to investigate the filler distribution‐electrical property relationship in composites with complex functional inorganic/organic fillers. The modified phase‐field model couples the charge behavior and breakdown evolution through the bridge effect of traps and synchronize the simulation in time and space. The introduction of organic molecules changes trap characteristics, leading to the redistribution of space electric field. The decreased electric field near the electrodes further delays the development of breakdown phase evolution and increases the breakdown strength, which is consistent with the experimental results. The expandability of this approach has been demonstrated in both organic‐ and inorganic‐filler reinforced composite dielectrics. Subsequently, high‐throughput calculations are carried out to optimize the designed bi‐gradient organic/inorganic structure. Moreover, a seven‐layer PI/BT/PCBM composite film with an interpenetrating gradient structure is prepared, exhibiting ultrahigh discharged energy density of 5.51 J cm^−3^ at 200 °C, which is far superior to the pure polymer and many other existing polymer composites. This work fills the gap in the study of breakdown process of composite dielectrics containing charge‐inhibiting components, and can be extended to the optimization and prediction of materials for other applications.

## Conflict of Interest

The authors declare no conflict of interest.

## Supporting information

Supporting Information

## Data Availability

The data that support the findings of this study are available from the corresponding author upon reasonable request.
