# Supplementary material for: A Bi‐Gradient Dielectric Polymer/High‐Κ Nanoparticle/Molecular Semiconductor Ternary Composite for High‐Temperature Capacitive Energy Storage
Source: Adv Sci (Weinh). 2023 Jul 14;10(26):2302949. doi: 10.1002/advs.202302949 (PMC10502658; doi:10.1002/advs.202302949)
Supplement: Supplementary file 1 — Supporting Information [file ADVS-10-2302949-s001.pdf]

ADVANCED SCIENCE
Open Access

Supporting Information

for *Adv. Sci.*, DOI 10.1002/advs.202302949

A Bi-Gradient Dielectric Polymer/High-K Nanoparticle/Molecular Semiconductor Ternary Composite for High-Temperature Capacitive Energy Storage

*Manxi Li, Yujie Zhu, Rui Wang, Jing Fu, Zhaoyu Ran, Mingcong Yang, Junluo Li, Jun Hu, Jinliang He and Qi Li**

Supporting Information

Rational design on dielectric polymer/high-*κ* nanoparticle/molecular semiconductor ternary composite for high-temperature energy storage

*Manxi Li, Yujie Zhu, Rui Wang, Jing Fu, Zhaoyu Ran, Mingcong Yang, Junluo Li, Jun Hu, Jinliang He, Qi Li**

Methods

Charge injection calculation: The bipolar charge injection model describes the distribution and the propagation of the free electrons, the free holes, the trapped electrons, and the trapped holes.[S2] The source item includes the combination of the particles and the processing for the transformation of free particles and trapped particles. Based on principle of the charge transportation, the control equations of the dipolar charge transmission can be expressed as:

$$\frac{dn_e}{dt} + \nabla\left(-n_e\mu_e\vec{E} - D_e\nabla n_e\right) = -R_{eh}n_en_h - R_{eht}n_en_{ht} - T_en_e\left(1-\frac{n_{et}}{n_{0et}}\right) + v\exp\left(\frac{\Phi_{et}}{kT}\right)n_{et}\frac{n_{et}}{n_{0et}} \quad (S1)$$

$$\frac{dn_h}{dt} + \nabla\left(-n_h\mu_h\vec{E} - D_h\nabla n_h\right) = -R_{eh}n_en_h - R_{eth}n_{et}n_h - T_hn_h\left(1-\frac{n_{ht}}{n_{0ht}}\right) + v\exp\left(\frac{\Phi_{ht}}{kT}\right)n_{ht}\frac{n_{ht}}{n_{0ht}} \quad (S2)$$

$$\frac{dn_{et}}{dt} = -R_{eth}n_{et}n_h - R_{etht}n_{et}n_{ht} + T_en_e\left(1-\frac{n_{et}}{n_{0et}}\right) - v\exp\left(\frac{\Phi_{et}}{kT}\right)n_{et}\frac{n_{et}}{n_{0et}} \quad (S3)$$

$$\frac{dn_{ht}}{dt} = -R_{eht}n_en_{ht} - R_{etht}n_{et}n_{ht} + T_hn_h\left(1-\frac{n_{ht}}{n_{0ht}}\right) - v\exp\left(\frac{\Phi_{ht}}{kT}\right)n_{ht}\frac{n_{ht}}{n_{0ht}} \quad (S4)$$

$$\nabla^2\phi = -\frac{e(n_h+n_{ht}-n_e-n_{et})}{\varepsilon_0\kappa} \quad (S5)$$

where E is the electric field, n_e and n_h represent the density of the free electron and free hole. n_{et} and n_{ht} represent the density of the trapped electron and trapped hole. R_{eh}, R_{eht}, R_{eth}, and R_{etht} represent the compound coefficient between the free electron and the free hole, the free electron and the trapped hole, the trapped electron and the free hole, the trapped electron, and the trapped hole. n_{0et} and n_{0ht} represent deep trap density for the electron and the hole. T_e and T_h represent the capture coefficient for the electron and the hole. v represents the coefficient for detaching the trap. Φ_{et} and Φ_{ht} represent the trap depth for the electron and the hole.

Charges can be injected from the electrodes and trapped by the charge traps at the particle/polymer interfaces, which induce electric field redistribution and influence the electrical tree propagation. The charge injection under high electric field and high temperature can be described from the Richardson-Schottky model, which is described as following.

$$J_{c,a} = AT^2 \exp\left(\frac{-\Phi_{c,a}}{kT}\right) \exp\left(\frac{e}{kT}\sqrt{\frac{eE_{c,a}}{4\pi\varepsilon_0}}\right) \tag{S6}$$

The charge flux $q_{ie,h}$ can be expressed as:

$$q_{ie,h} = j_{c,a}/e \tag{S7}$$

The charge diffusion factor D and the charge mobility μ can be expressed as:

$$D = \mu\frac{kT}{e} \tag{S8}$$

$$\mu = \mu_0 \exp\left(\sqrt{\frac{e^3 E}{4\pi\varepsilon}}/kT\right) \tag{S9}$$

where $J_{c,a}$ are the Richardson-Schottky current density on the cathode and the anode, A is the Richardson coefficient. $\Phi_{c,a}$ are the injection barrier for the cathode and the anode, m is the electron mass, k is the Boltzmann constant, T is the absolute temperature, ε is the dielectric constant. The bipolar charge injection model is simulated in FEM (Finite Element Method) on COMSOL Multiphysics in 1d coordinate. The values of these parame- ters are listed in Table S1.

Table S1. The material parameters used in bipolar charge injection.

Parameters	Values
$\mu_{e,h}$ / (m^2 V^{-1} s^{-1})	4×10^{-15}, 4×10^{-16}
$R_{eht,eth,etht}$ / (m^3 s^{-1})	6.4×10^{-22}
R_{eh} / (m^3 s^{-1})	0
$n_{0et,ht}$ / (m^{-3})	6.3×10^{20}
$T_{e,h}$ / (s^{-1})	0.1, 0.1
$\Phi_{et,ht}$ / (eV)	0.95, 0.95
$\Phi_{c,a}$ / (eV)	1.2, 1.2
V / (s^{-1})	6×10^{12}
κ	3.2

Phase field modeling: After solving the bipolar charge injection model on COMSOL Multiphysics, the calculated charge density will be turned into the phase field model, which will affect the electrostatic free energy, and affect the phase parameter η(r) path growth afterwards. In the phase field model, the free energy F contains electrostatic energy f_{sep}, distribution energy f_{grad}, gradient energy f_{elec}, thermal energy f_{Joule}, and tensile energy f_{strain}.

$$F = \int [f_{sep}(\eta(\boldsymbol{r})) + f_{grad}(\eta(\boldsymbol{r})) + f_{elec}(\eta(\boldsymbol{r})) + f_{Joule}(\eta(\boldsymbol{r})) + f_{strain}(\eta(\boldsymbol{r}))]\, dV \tag{S10}$$

Where this free energy can be expressed as:

$$f_{grad} = -\frac{1}{2}\gamma|\nabla\eta(\boldsymbol{r})|^2 \quad \text{(S11)}$$

$$f_{sep} = \alpha\eta^2(1-\eta)^2 \quad \text{(S12)}$$

$$f_{elec} = \frac{1}{2}\varepsilon_0\varepsilon_{ij}E^2 \quad \text{(S13)}$$

$$f_{Joule} = \sigma_{ij}E^2dt \quad \text{(S14)}$$

$$f_{strain} = \frac{\sigma_m^2}{2Y} = \frac{\varepsilon_0^2\varepsilon_{ij}^2E^4}{8Y} \quad \text{(S15)}$$

where the γ is the gradient energy coefficient, α is a positive coefficient defining the energy barrier of the phase separation, ε is the permittivity of different components, E is the electrical field strength distribution in the simulation area, σ_{ij} is the spatially and temperature dependent electrical conductivity tensor, dt is the operating time of applied electric field, σ_m is the mechanical compressive stress, Y represents the Young's modulus.

Table S2. The input parameters of PI/PCBM composites used in modified phase-field modeling.

Parameters	Phase	Values
dielectric constant κ	PI	3.2
	PCBM	3.9
electrical conductivity σ (S m^{-1})	PI	$\log\sigma = 3.57 - 6.38 \times 1000/T$
	PCBM	0.015
intrinsic breakdown strength E_b (MV m^{-1})	PI	$450 - 1.14 \times (T - 298)$ (MV m^{-1})
	PCBM	-
trap depth (eV) and density (10^{20}/eV m^3) of composite with different volume fraction of PCBM	0.25 vol%	1.39 and 10.5
	0.5 vol%	1.41 and 22.8
	0.75 vol%	1.4 and 18.3
	1 vol%	1.4 and 15.3

A modified Allen-Cahn equation is employed to describe the breakdown phase evolution,

$$\frac{\partial\eta(\boldsymbol{r},t)}{\partial t} = -L_0H(f_{elec} + f_{Joule} + f_{strain} - f_{critical})[\frac{\partial f_{sep}}{\partial\eta} + \frac{\partial f_{grad}}{\partial\eta} + \frac{\partial f_{elec}}{\partial\eta} + \frac{\partial f_{Joule}}{\partial\eta} + \frac{\partial f_{strain}}{\partial\eta}] \quad \text{(S16)}$$

Where L_0 is the kinetic coefficient relating to the interface mobility with a value of 1 m^2 s^{-1} N^{-1}, and H is the Heaviside unit step function. [S3]

The phase field model is written in Python code in 2D XY coordinate.

Experimental part:

Synthesis of composites: 4,4-Diaminodicyclohexyl methane and 4,4'-(hexafluoroisopropylidene) diphthalic anhydride were purchased from Anhui Zesheng Technology Co., Ltd. BT nanoparticles, BNNS and the molecular semiconductor PCBM were all purchased from Sigma-Aldrich, and they were used without further purification. Firstly, a certain mass of dianhydride monomer was added to NMP solution. Then, an equal molar amount of diamine monomer was added to the mixed solution, followed by stirring at 80 °C for 12 h to ensure that the diamine and the dianhydride monomer reacted to form a poly(amic acid), as shown in **Figure S2**. Next, corresponding mass fraction of BT or PCBM fillers were weighted and fully dispersed in NMP solvent by stirring and ultra- sonication for 1 h to dissolve well. Subsequently, they were added into the reacted PI solution and continued to stir for 12 h so that the obtained solution was homogeneous. Afterwards the reacted solution was cast on a pre-cleaned glass plate and dried in an oven by a gradient heating method. The composites were heated at 80 °C for 12 h, then 150 °C, 200 °C, and 250 °C in sequence for 1 h to remove solvent completely. A layer-by-layer solution casting method was performed by repeating above steps several times to obtain multiple layers composites. Finally, the flexible films were peeled off from the glass substrate.

Characterization: Scanning electron microscopy (SEM) were conducted on a ZEISS MERLIN Compact field emission electron microscope. A Novocontrol Concept 80 dielectric spectroscopy meter equipped with a Quatro-Cryosystem temperature control system was used for obtaining dielectric constant and dissipation factor, the electrode diameter used in this test was 10 mm. Dielectric breakdown strength was recorded on a TREK 610C amplifier at a voltage rise rate of 500 V s^{-1}, 15 data were obtained for each sample. Circular gold electrodes with a diameter of 3 mm and a thickness of 60 nm were sputtered on the two sides of the samples before electrical measurements. In the dielectric breakdown strength and leakage current tests, the electrode diameter of the sample was 2 mm. The D-E loops were recorded on a modified Sawyer-Tower circuit under a unipolar wave at the frequency of 100 Hz, and the electrode diameter used in this test was 3 mm. In the breakdown strength and D-E loops tests, the testing samples were soaked in dimethylsiloxane, and the temperature of silicone oil was controlled by a hot plate equipped with a temperature sensor. TSDC tests were measured by a Keithley 6517B amperemeter equipped with

a Quatro-Cryosystem temperature control system. First, all samples were polarized under 50 MV m^{-1} DC poling electric field for 30 min at 200 °C, then quickly cooled to 30 °C with the electric field remaining 50 MV m^{-1}. Finally, removed the electric field and short-circuit the sample after holding at 30 °C for 10 min, heated the sample to 250 °C at a heating rate of 3 °C min_{-1}, and recorded the current at each temperature point. The results were analyzed using the half-peak method after peak fitting by Equation S17 and S18. [S4]

$$Q_{\mathrm{TSDC}} = \frac{60}{\beta} \int_{T_0}^{T_1} I(T) dT \tag{S17}$$

$$E = \frac{2.47 T_{\mathrm{m}}^2 k}{\Delta T} \tag{S18}$$

where β is the heating rate, T_0 and T_1 mean the starting and ending temperatures of a peak respectively, $I(T)$ is the TSDC curves, T_m describes the temperature *corresponding* to the peak position, ΔT is the difference in temperature corresponding to the half peak and k is the Boltzmann constant.

Statistical Analysis: The Weibull breakdown strength is studied based on the function $P = 1 - \exp((E_{\mathrm{b}}/E_0)\beta)$, where P describes the cumulative probability of dielectric breakdown, E_0 is the characteristic breakdown strength that corresponds to 63.2% probability of failure, E_b is the experimental breakdown field strength and the shape parameter β reflects the reliability of materials that evaluate the scatter of data. There are 14 samples for each statistical analysis, the maximum and minimum values are eliminated, with 12 samples left. Statistical analysis was carried out using Origin Software.

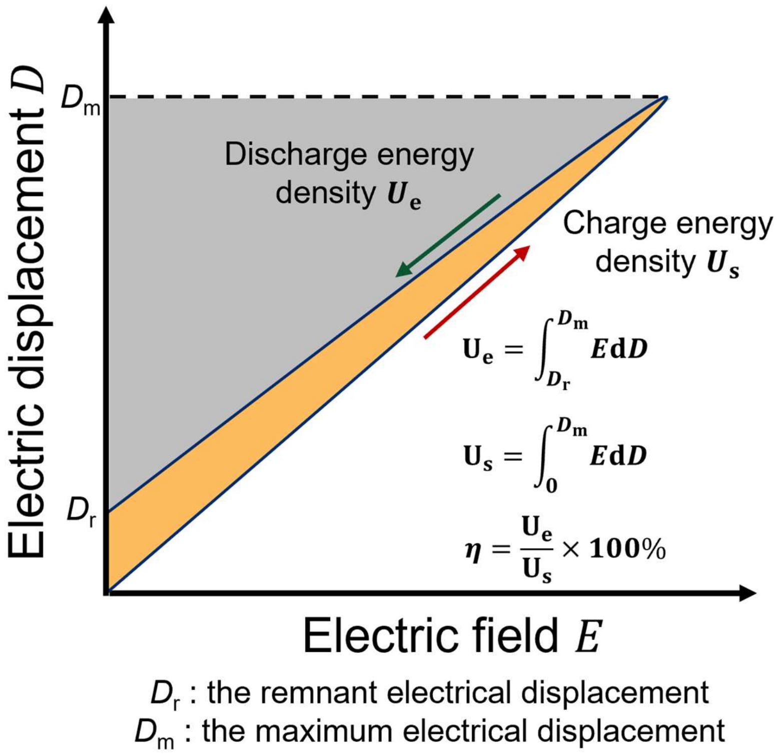

Figure S1. Schematic diagram of calculating the energy density of a dielectric.

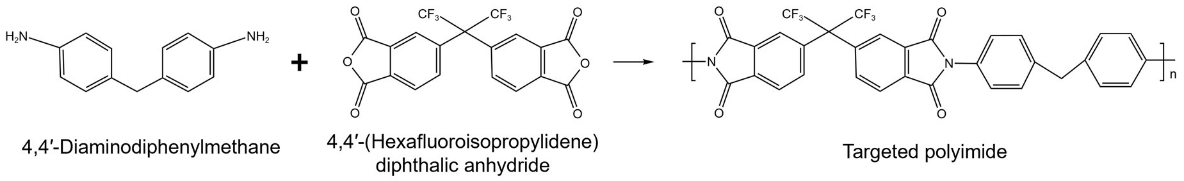

Figure S2. Schematic diagram of the synthesis process of the PI .

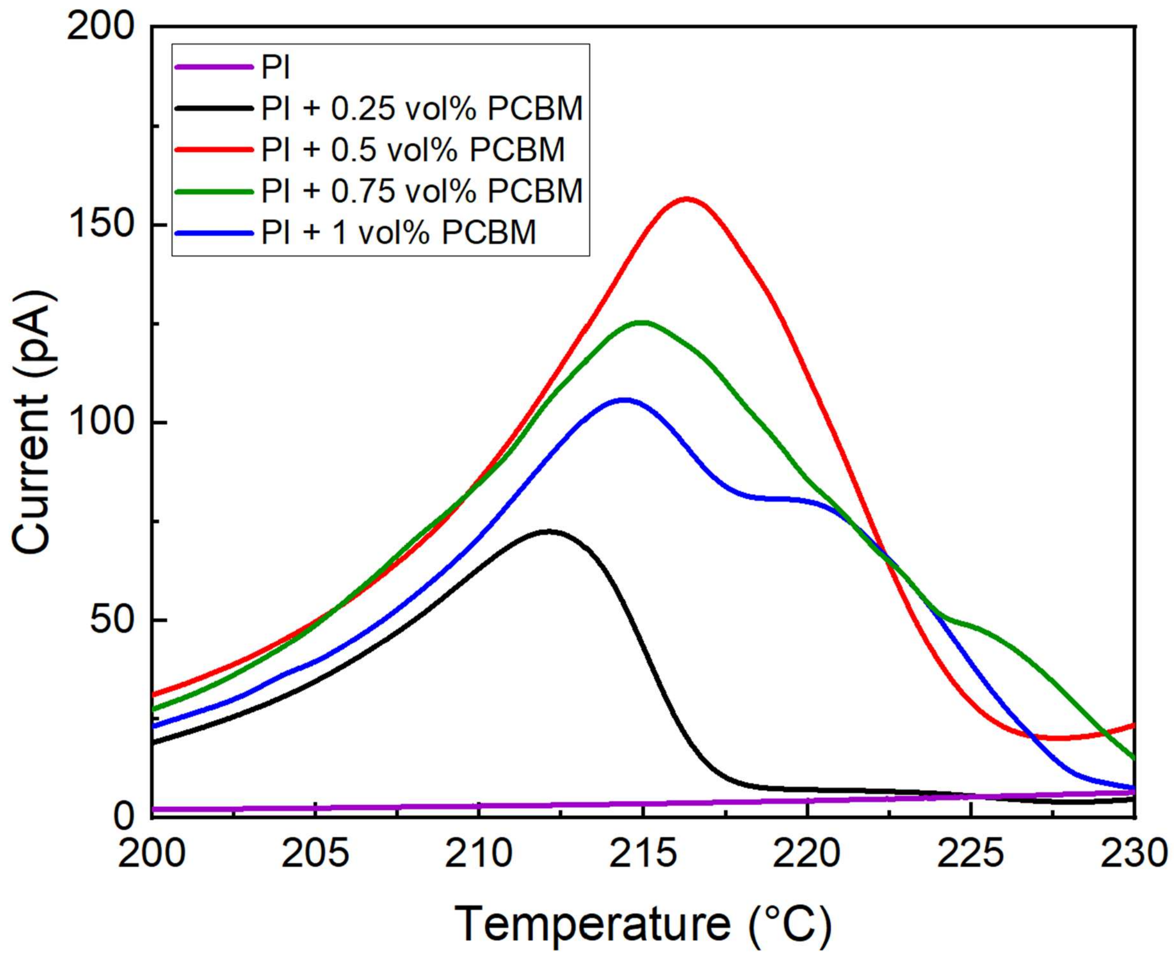

Figure S3. TSDC spectra of PI and the PI/PCBM composites.

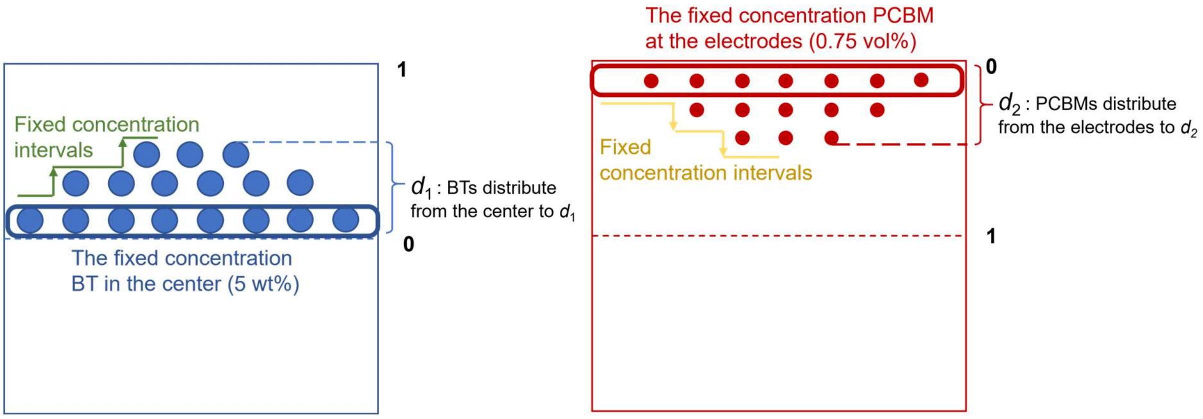

Figure S4. The parameter diagram of the designed bi-gradient structure.

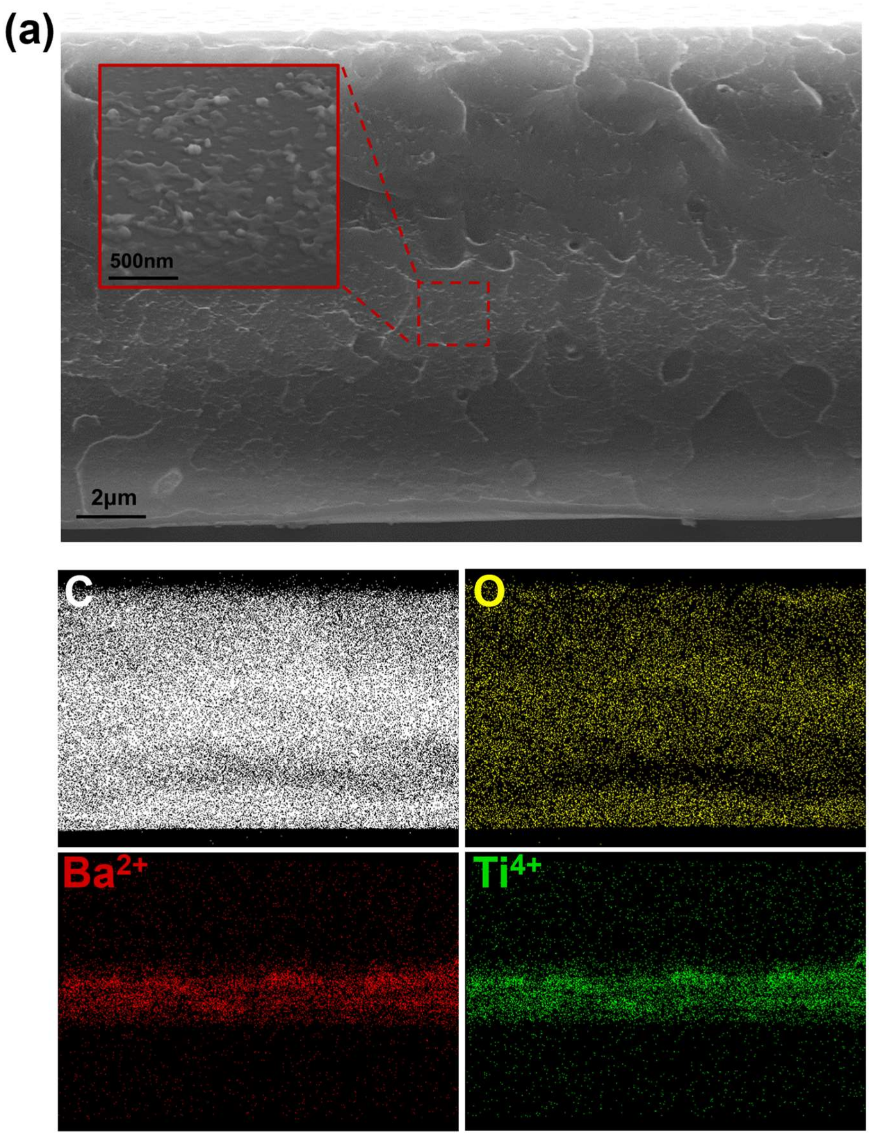

Figure S5. (a) Cross-sectional SEM images and (b) EDS mapping analysis of the composite film with distribution of 0.75%/6L-2.5%/1L.

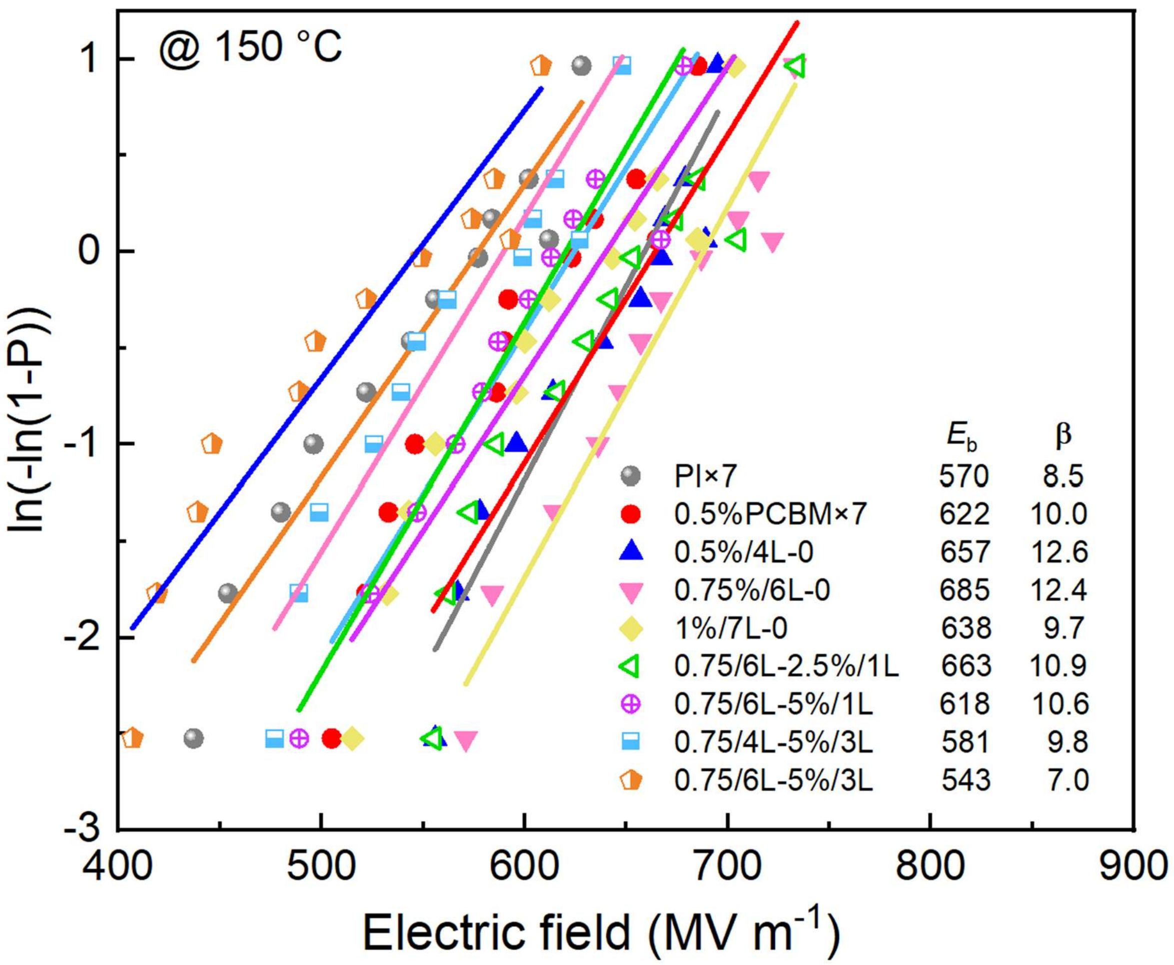

Figure S6. Weibull breakdown strength at 150 °C for composites with different structures.

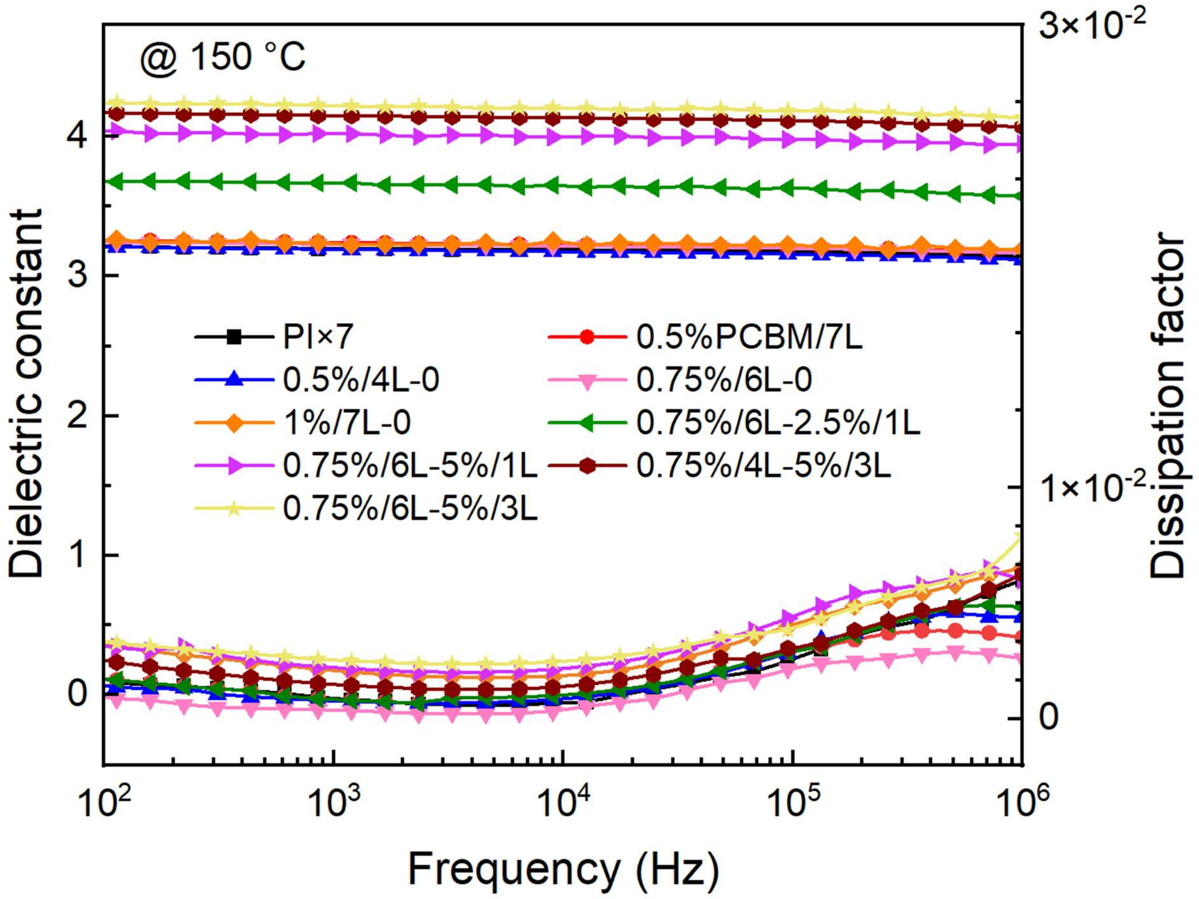

Figure S7. Dependencies of dielectric constant and dielectric loss on frequency at 150 °C for composites with different structures.

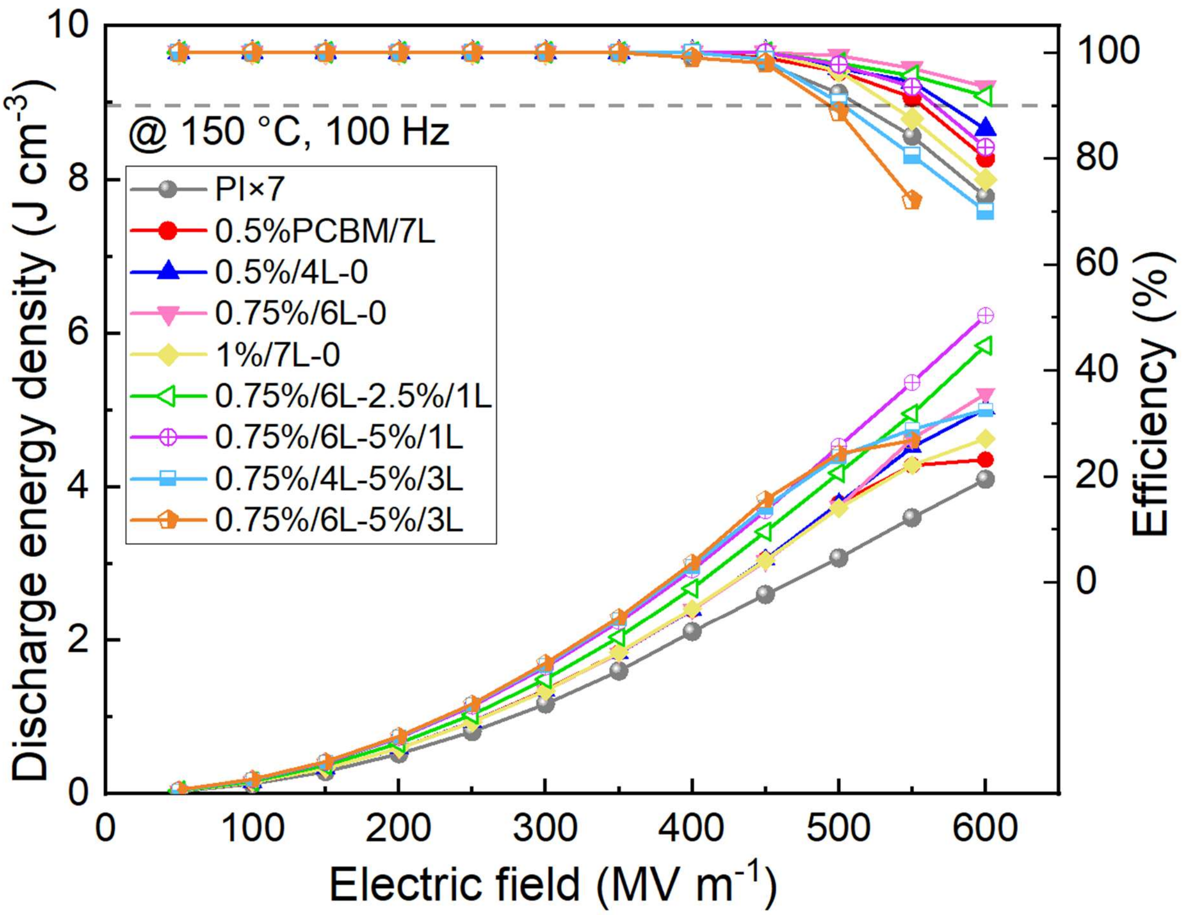

Figure S8. Discharged energy density and charge-discharge efficiency at 150 °C and 100 Hz for composites with different structures.

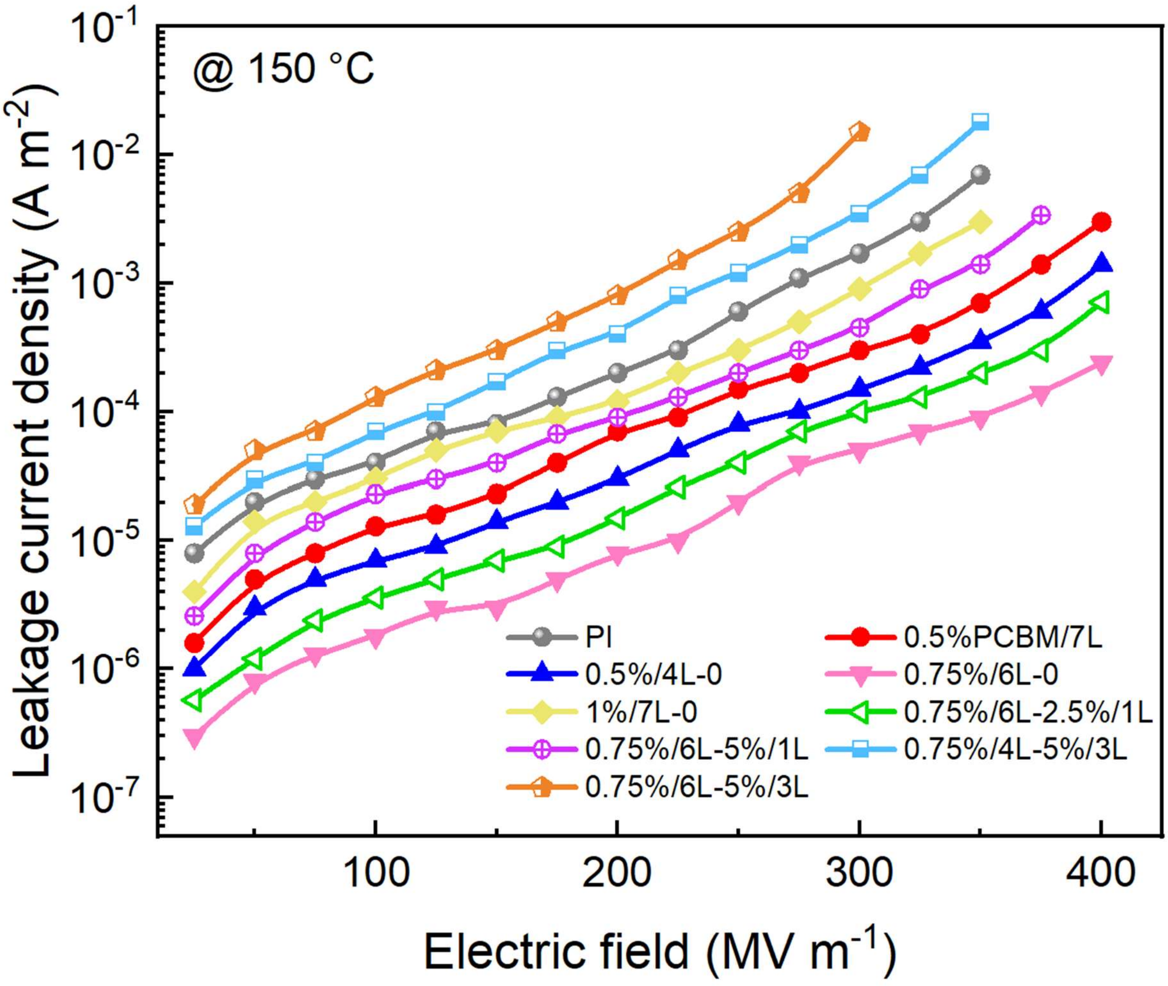

Figure S9. Electric field-dependent leakage current density at 150 °C.

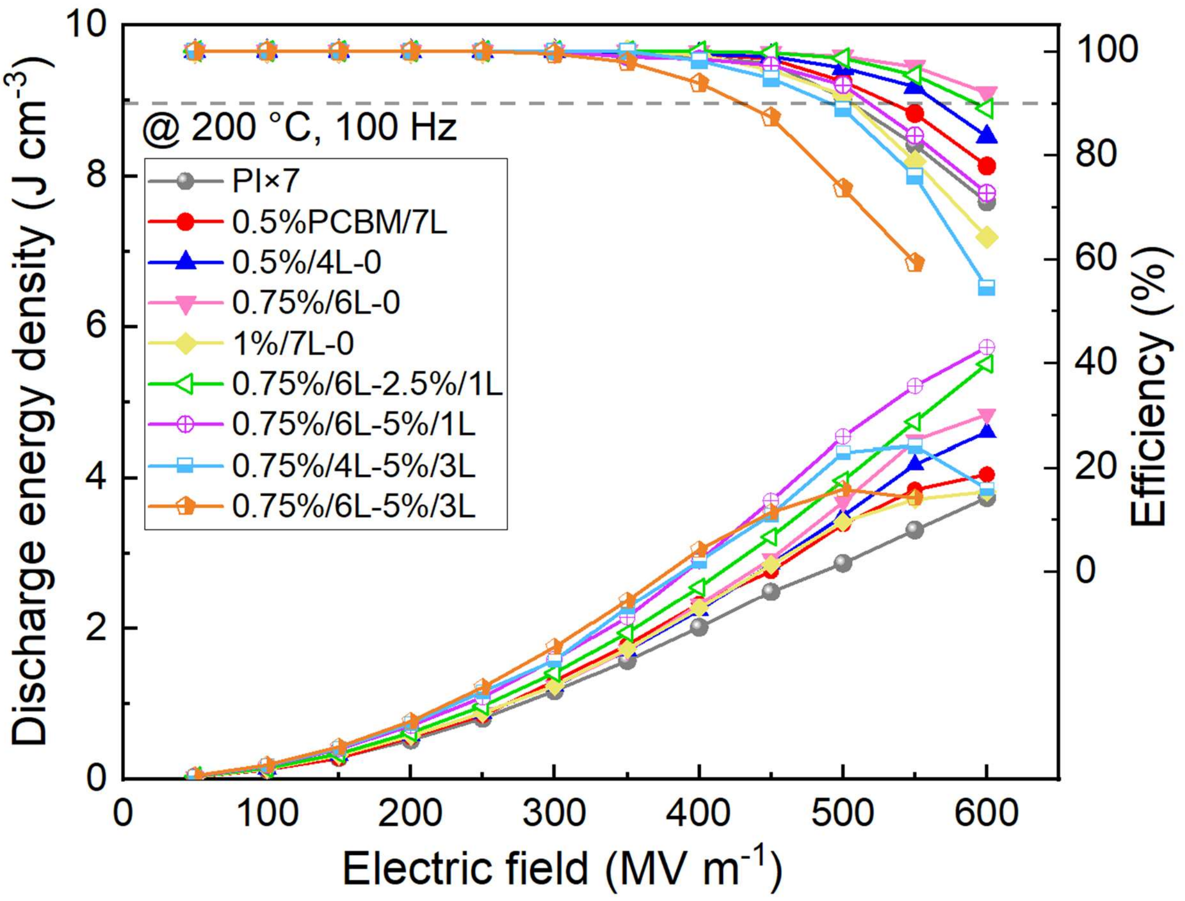

Figure S10. Discharged energy density and charge-discharge efficiency at 200 °C and 100 Hz for composites with different structures.

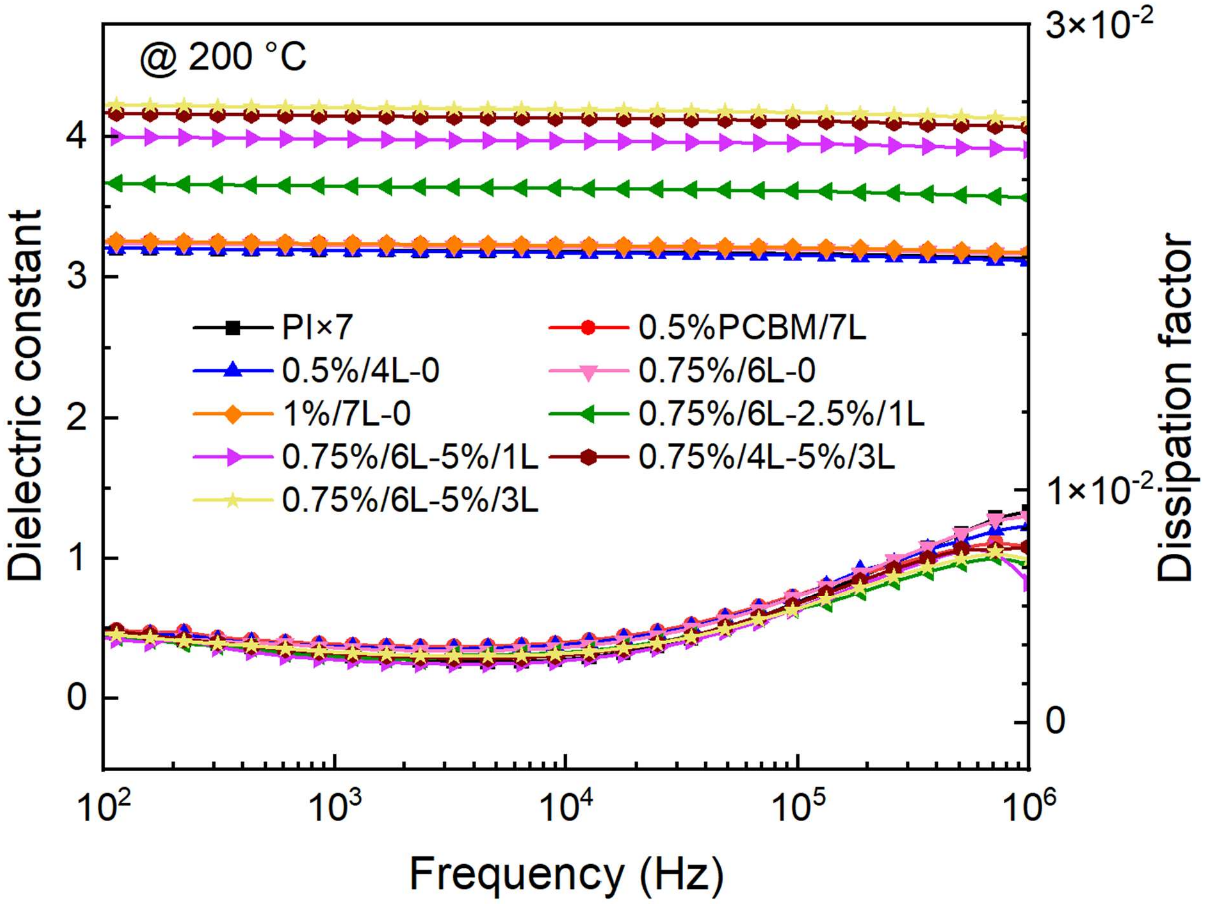

Figure S11. Dependencies of dielectric constant and dielectric loss on frequency at 200 °C for composites with different structures.

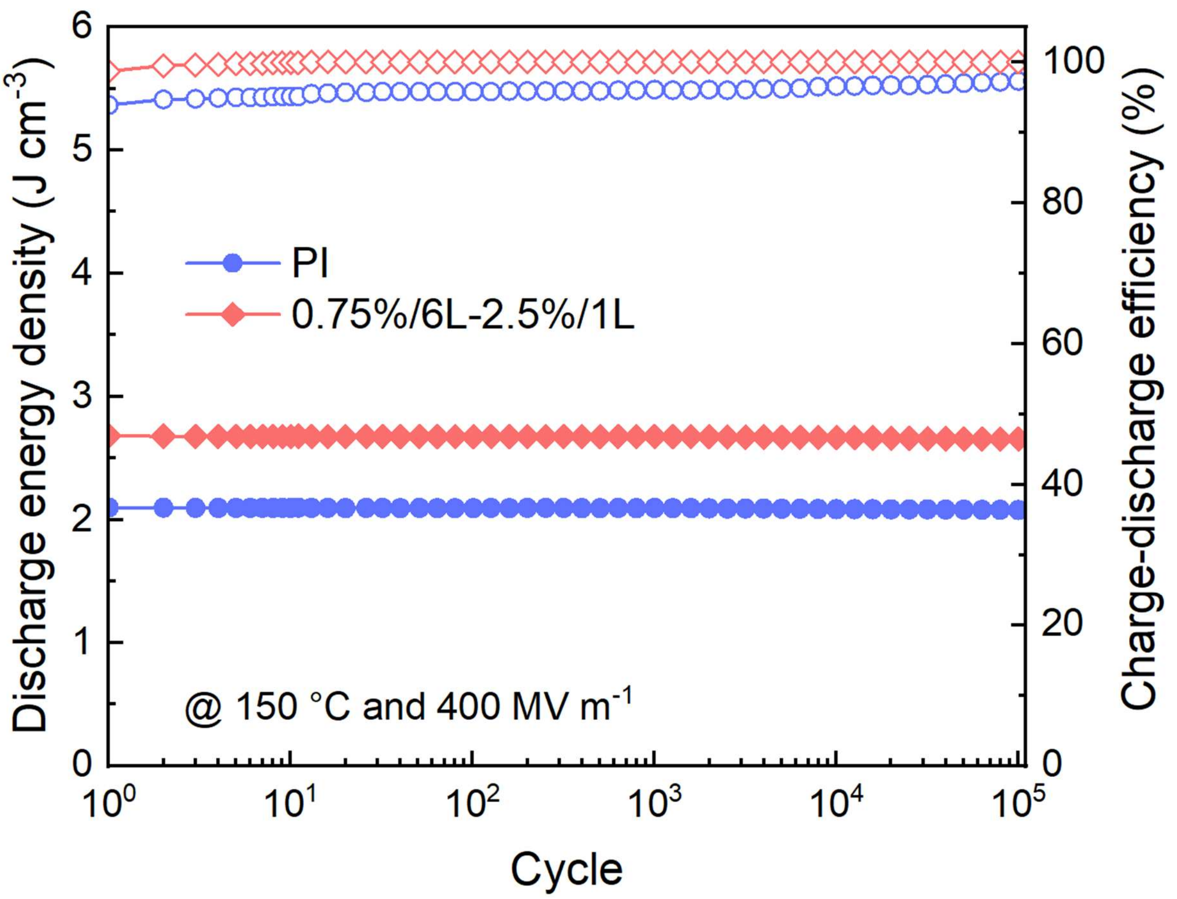

Figure S12. Cyclic stability of PI and the 0.75%/6L-2.5%/1L composite at 150 °C and 400 MV m^{-1}.

[S1] R. A. Marcus, N. Sutin, *Biochimica et Biophysica Acta (BBA)-Reviews on Bioenergetics* **1985**, *811*, 3 265

[S2] J. Alison, R. Hill, *Journal of Physics D: Applied Physics* **1994**, *27*, 6 1291.

[S3] Z.-H. Shen, J.-J. Wang, J.-Y. Jiang, S. X. Huang, Y.-H. Lin, C.-W. Nan, L.-Q. Chen, Y. Shen, *Nature Communications* **2019**, *10*, 1 1.

[S4] S. Li, D. Min, W. Wang, G. Chen, *IEEE Transactions on Dielectrics and Electrical Insulation* **2016**, *23*, 5 2777.
